# Platelet-derived NO slows thrombus growth on a collagen type III surface

**DOI:** 10.1186/1477-9560-2-11

**Published:** 2004-11-15

**Authors:** Robert H Williams, Matthias U Nollert

**Affiliations:** 1 University of Oklahoma, School of Chemical Engineering and Material Science, 100 East Boyd, Norman, OK 73019, USA

## Abstract

Nitric oxide (NO) is a free radical that plays an important role in modulating platelet adhesion and aggregation. Platelets are a source of vascular NO, but since erythrocytes avidly scavenge NO, the functional significance of platelet-derived NO is not clear. Our purpose was to determine if NO from platelets affects platelet thrombus formation in the presence of anticoagulated whole blood in an *in vitro *parallel plate flow system. We studied platelet adhesion and aggregation on a collagen type III surface in the presence of physiologically relevant fluid mechanical shear stress. We found that certain receptor mediated agonists (insulin and isoproterenol) caused a concentration dependent reduction in thrombus formation at a shear rate of 1000 s^-1^. This effect was mediated by NO since it was abolished in the presence of the NO inhibitor L-nitro-arginine-methyl-ester (L-NAME). As expected, at venous levels of shear rate (100 s^-1^) neither of the agonists had any effect on thrombus formation since platelet adhesion does not depend on activation at these low levels of shear. Interestingly, at a shear rate of 2000 s^-1 ^the addition of L-NAME caused an increase in platelet coverage suggesting that shear, by itself, induces NO production by platelets. This is the first demonstration of shear stress causing platelets to produce an inhibitor of platelet activation. These results demonstrate that the development of a platelet thrombus is regulated in a complex way and that platelets produce functionally significant amounts of NO even in the presence of whole blood.

## Introduction

Platelet activation and aggregation play an important role in the development of cardiovascular disease, which is the leading cause of death in the United States. Over the last several years, enormous advances have been made in understanding the molecular mechanisms that regulate platelet function. A general paradigm has emerged that endothelial cells synthesize and release substances that inhibit platelet activation (e.g. nitric oxide and prostacyclin) except near a site of vascular injury, while platelets synthesize and release substances that promote further platelet activation. In contrast to this paradigm, several recent studies have suggested that, in the presence of certain agonists (IGF-1, adenosine diphosphate, insulin, and isoproterenol), platelets will produce nitric oxide (NO), a potent inhibitor of platelet activation [1-5]. Although these studies clearly showed that platelets produce NO, the physiological relevance of this NO is not clear since NO is highly reactive, has a short half-life, and the rate of NO production is difficult to quantify. Therefore, there is a need to determine if the NO derived from platelets is sufficient to modulate platelet function and alter thrombus growth in the presence of whole blood.

Platelet-derived NO is synthesized by membrane-bound endothelial-type nitric oxide synthase (eNOS). A common signaling mechanism shared by many eNOS agonists is binding to a surface receptor, followed by activation of phosphatidylinositol-3-kinase (PI3K), which results in phosphorylation of eNOS at serine 1179 through Akt [6-8]. The activated eNOS converts L-arginine to L-citrulline and produces NO as a result [9]. Nitric oxide is a water soluble free radical that can bind to the heme-soluble site of guanylate cyclase in platelets and smooth muscle cells, which increases synthesis of cyclic guanosine monophosphate (cGMP) [5,10]. cGMP can bind to phosphodiesterase III (PDE III), which reduces metabolism of cyclic adenosine monophosphate (cAMP) [10]. Elevated levels of cGMP and cAMP can result in increased activity of protein kinase G (PKG) and protein kinase A (PKA), which inhibit protein kinase C (PKC) activation and intracellular Ca^2+ ^mobilization [5]. The consequence of this signal transduction cascade is the inhibition of platelet activation and the relaxation of vascular smooth muscle resulting in blood vessel dilation.

As a small, hydrophilic free radical NO is highly diffusible in the aqueous environment of the blood. However, it is also highly reactive with a very short half-life estimated to be only on the order of a few seconds [11] in the blood. Previous studies that demonstrated NO production in platelets were done in the absence of erythrocytes [2-4]. It is still not clear if the amount of NO produced by platelets, estimated at about 5 × 10^-17 ^mole NO/platelet (determined by microelectrode in PRP for 2 minutes following addition of 5 μM ADP), is sufficient to alter platelet function [1,2].

The purpose of our study was to determine if platelet-derived NO plays a role in thrombus formation in the presence of shear stress. We examined the effect of several external factors on platelet thrombus formation, including insulin, the β-adrenoceptor agonist isoproterenol, and shear stress. Previous studies showed that insulin and isoproterenol both induced NO formation by platelets [3,4]. Shear stress is an important component of the environment of the platelets and can cause alterations in platelet function, although the effect of shear on NO synthesis in platelets is unknown [12]. Our aim was to show that platelet-derived NO plays a direct role in inhibiting thrombus formation to a vascular injury and can be stimulated by different external agonists through a common NO signaling pathway.

## Results

### Insulin and isoproterenol slow the growth rate of mural thrombi

**Figure 1:**
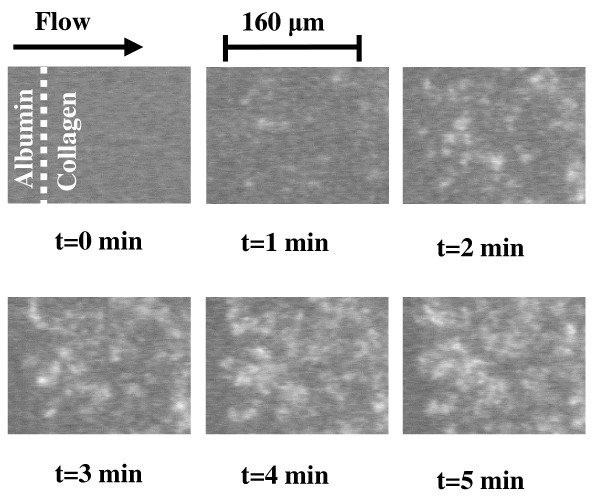
Representative images of time-dependent platelet adhesion and aggregate formation on collagen type III. Single platelets and platelet aggregates adherent on the surface appear bright and were visualized using epi-fluorescence video microscopy. Platelets adhered abundantly on the collagen surface, particularly near the upstream interface between collagen and albumin. No adhesion was observed on the albumin-coated surface. Flow was from left to right and the shear rate was 1000 s^-1^. These results are typical of 5 separate experiments.

**Figure 2:**
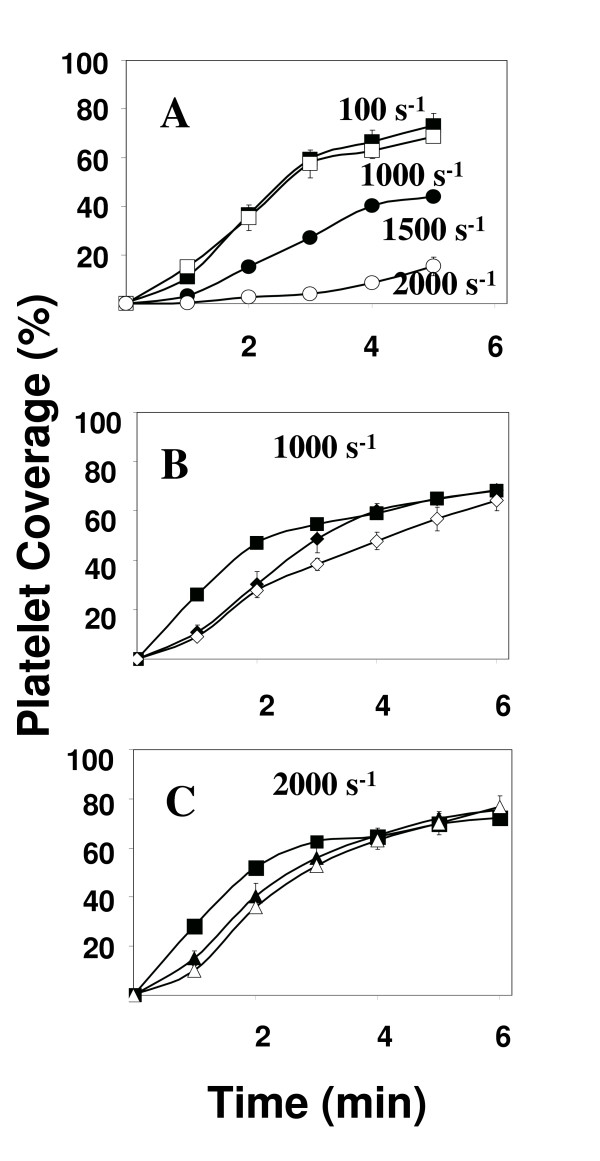
Dose dependence of thrombus formation. The extent of platelet adhesion onto the surface, as assessed by the percentage of the collagen-coated surface covered by platelets, is shown as a function of time. Perfusions for shear [100 (■), 1000 (□), 1500 (●), and 2000 (○) s^-1^] were performed for 5 minutes (Figure 2A). Perfusions for control (■), insulin [100 (◆) and 1000 pM (◇)] and isoproterenol [100 (▲) and 1000 μM (△)] were performed at 1000 s^-1 ^(Figures 2B-C) for 6 minutes. The results presented are mean ± s.e.m. of at least 5 experiments using 5 different blood donors.

Platelets from whole blood adhered avidly to the collagen-coated surface in the presence of physiologically relevant levels of fluid mechanical shear stress. Very little, if any, platelets were detected on the albumin-coated portion of the slide. Detectable levels of platelet adhesion were evident within 20–30 seconds of the initiation of blood flow over the surface. Platelet adherence occurred predominantly at the interface between albumin and collagen and increased as a function of time similar to results that have been obtained by others using a similar system [13-16] Representative images of platelet accumulation on collagen at a shear rate of 1000 s^-1 ^are shown in Figure 1. In these images, the flow was from left to right. The albumin/collagen interface is clearly evident in the images from later time points. At other shear rates, platelet accumulation on the surface was also abundant as illustrated in Figure 2A. These results are in qualitative agreement with previously published results [13]. The slower rate of platelet deposition at higher levels of shear has been attributed [17] to the increased level of fluid mechanical drag on the platelets preventing them from forming stable attachments to the surface.

The effect of insulin and isoproterenol on mural thrombus formation was studied and the results are presented in Figures 2B and 2C as the percent coverage of the collagen-coated surface as a function of time. Increasing the concentration of either insulin (0, 100, 1000 pM) or isoproterenol (0, 100, 1000 μM) at a shear rate of 1000 s^-1 ^resulted in increasing inhibition of platelet accumulation on the surface. Higher concentrations of either insulin or isoproterenol had no additional affect on the extent of platelet accumulation on the surface (data not shown). Inhibition of thrombus formation was significant (p < 0.05) up to 6 minutes with 1000 pM insulin and up to 3 minutes with 1000 μM isoproterenol. Significance was determined with SPSS using the Post Hoc test as described in Methods.

### Agonist induced reduction in thrombus formation depends on NO and shear rate

**Figure 3:**
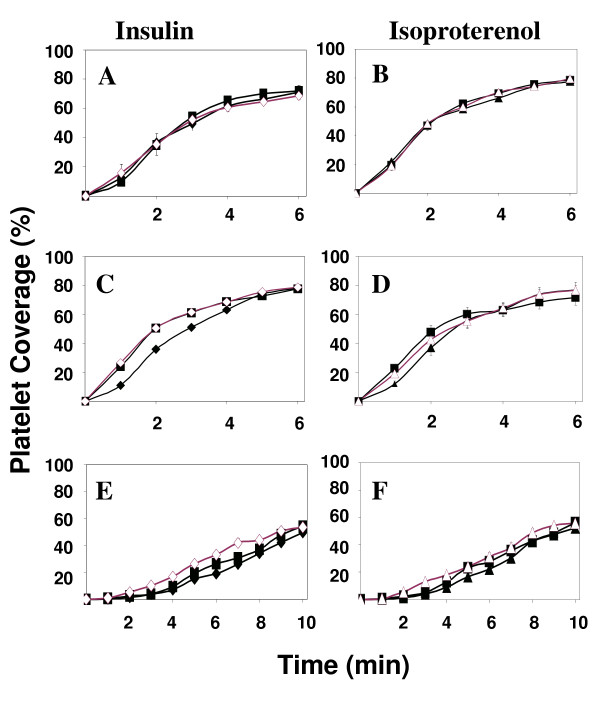
Reduction in thrombus growth rate depends on platelet derived NO. Perfusions for control (■), insulin, 500 pM (◆), isoproterenol, 100 μM (▲), and samples preincubated with L-NAME [insulin (◇) and isoproterenol (△)] were performed at 100, 1000, and 2000 s^-1 ^(Figures 3A-F). Results represent mean ± s.e.m. of at least five experiments with five separate blood donors.

In order to investigate the mechanism of insulin and isoproterenol induced reduction in platelet accumulation on a collagen surface, we perfused blood through the flow chamber in the presence of agonist (either insulin or isoproterenol) as well as L-NAME, a specific inhibitor of nitric oxide production. The results are presented in Figures 3C,3D for the shear rate of 1000 s^-1^. In the presence of L-NAME as well as either insulin or isoproterenol, thrombus formation is increased, returning to levels seen in the absence of added agonist. This suggests a role for nitric oxide in mediating the agonist induced reduction in platelet accumulation on the surface. Platelet thrombus formation on the surface at lower (100 s^-1^) and higher (2000 s^-1^) levels of shear rate are shown in Figures 3A,3B and 3E,3F respectively. At the lower, venous shear rate (100 s^-1^) platelet accumulation on the surface is not significantly altered by either insulin (500 pM) or isoproterenol (100 μM). Higher concentrations of either agonist had no affect as well (data not shown). In addition, L-NAME by itself or in combination with either agonist did not significantly affect platelet deposition on the surface (Figures 3A,3B). At the higher, arterial shear rate of 2000 s^-1 ^(Figures 3E,3F), platelet accumulation was not significantly reduced by either insulin (500 pM) and isoproterenol (100 μM) as illustrated in Figures 3E,3F.

**Figure 4:**
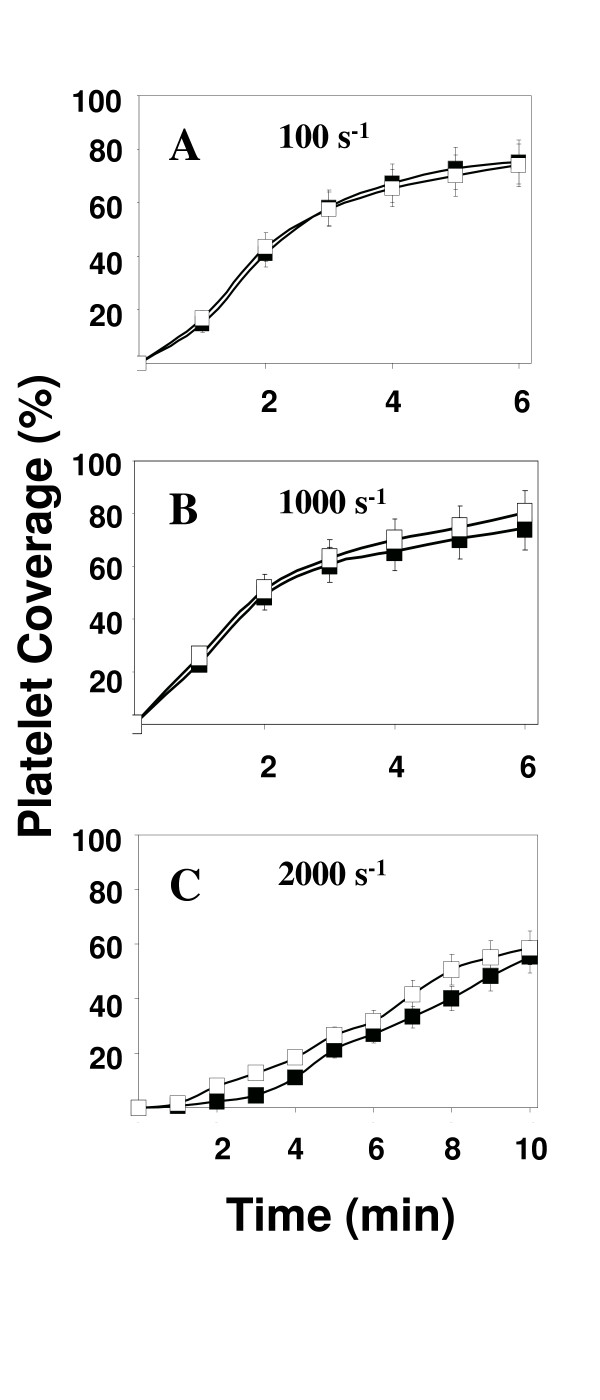
Shear stress causes a slowing of thrombus growth rate that depends of platelet derived NO. Platelet coverage values for each study are the mean ± s.e.m. of 5 separate experiments, each using a different donor. Perfusions for control (■) and L-NAME (□) samples were conducted at 100, 1000, and 2000 s^-1 ^(Figures 4A-C) for 6–10 minutes.

At the high shear rate of 2000 s^-1 ^we observed a curious result when the blood was treated with L-NAME but in the absence of any agonist. The results are presented in Figure 4. We found that in the presence of L-NAME, thrombus formation increased either with (data not shown) or without agonist present (Figure 4C). This result suggests a role for nitric oxide in modulating the rate of platelet deposition onto a collagen surface at a shear rate of 2000 s^-1 ^even in the absence of insulin or isoproterenol.

### Effect of insulin and isoproterenol on guanylate cyclase activity

**Figure 5:**
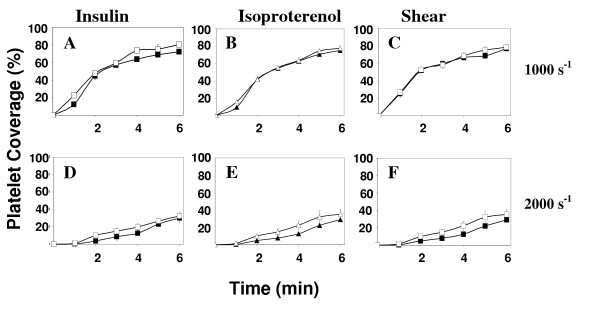
Effect of guanylate cyclase on platelet adhesion kinetics. Platelet coverage values for each study are the mean ± s.e.m of 4 separate experiments, each using a different donor. Perfusions were conducted at 1000 and 2000 s^-1 ^(Figures 5A and 5B) for 6 minutes with insulin, 500 pM (■), isoproterenol, 1000 μM (▲) and samples preincubated with guanylate cyclase inhibitor ODQ [insulin (□) and isoproterenol (△)].

We further investigated the mechanism of platelet derived nitric oxide inhibition of thrombus formation by using the guanylate cyclase inhibitor 1H-[1,2,4]oxadiazolo [4, 3-a]quinozalin-1-one (ODQ), since the primary target of NO in platelets is guanylate cyclase [18,19]. As illustrated in Figure 5A, thrombus formation at 1000 s^-1 ^and in the presence of insulin was slightly increased by addition of ODQ. No significant effect of ODQ on thrombus formation was observed with isoproterenol (Figure 5B) or in the absence of agonist (Figure 5C). This is not surprising since our other studies at this shear with isoproterenol (Figure 2D) or in the absence of agonist (Figure 3B) pointed out that the effect on platelet thrombus formation was also very small. Interestingly, we see that ODQ causes an increase in thrombus formation at 2000 s^-1 ^if either insulin, isoproterenol, or no agonist is used (Figures 5D,5E,5F). This suggests that at 2000 s^-1^, guanylate cyclase activation is occurring even in the absence of added external agonists.

## Discussion

The purpose of this study was to determine if nitric oxide from platelets affects platelet function. Previous studies have identified the vascular endothelium as the primary source of NO in the blood. Only recently have platelets been identified as an additional source of NO. The role of platelet derived NO in modulating platelet function has not been established. Most of the other studies that have examined NO production by platelets were performed with platelet rich plasma or with washed platelets. This was required in order to increase the platelet concentration to the point where NO levels could be assessed. However, since these studies were performed in the absence of erythrocytes, it is difficult to extrapolate the results to the *in vivo *situation where hemoglobin in red cells efficiently scavenges the nitric oxide free radical. Therefore, the experiments in our current study were designed to measure platelet function in a vascular injury model where the platelets remain in whole blood.

Several recent studies have demonstrated that platelets produce NO in response to agonists including insulin, isoproterenol, and ADP [2,4]. Using an *in vitro *parallel plate flow chamber, we show in this study that the rate of mural thrombus growth on a collagen III surface is modulated by platelet derived NO. This is the first study to show that platelet derived NO can alter platelet function in whole blood. We found that insulin, isoproterenol, and shear stress could, under the right circumstances, modulate platelet function through an NO-mediated mechanism. In fact, we found that at a shear rate of 2000 s^-1^, but not at either 100 or 1000 s^-1^, platelet adhesion to the surface was increased in the presence of L-NAME, an inhibitor of nitric oxide production. This result suggests that platelets produce nitric oxide at higher shear rates but not at lower levels of fluid mechanical stress. This result is consistent with the observation that elevated levels of shear stress can cause platelet activation [20,21] and that nitric oxide production may be a common feature of platelet activation regardless how activation is initiated [8,22].

At the low, venous level of shear (100 s^-1^) that we tested, platelet derived NO did not appear to play a role in modulating platelet accumulation on the surface. This result was not surprising due to the many types of adhesion receptors on platelets that function well in a low shear environment. The primary platelet receptors for collagen III on a surface are GPVI and α_2_β_1 _[23,24]. Additionally, the GPIb-V-X receptor complex can mediate platelet adhesion through interactions with soluble and surface associated von Willebrand Factor [25]. Because of the interaction with collagen, the platelets become activated. Activation induces an extensive series of intracellular signaling events to take place, resulting in the conversion of the integrins α_2_β_1 _and α_IIb_β_3 _into a high affinity state through a process termed inside-out signaling. In this high affinity state, these integrins are capable of supporting platelet-platelet interactions and promoting thrombus growth. Therefore, platelet adhesion at low levels of shear rate can be mediated by a number of different adhesion molecules, some of which do not require platelet activation. Our results are consistent with this model since we found that platelet derived nitric oxide did not inhibit the rate of thrombus growth at these low, venous levels of shear rate.

At intermediate shear (1000 s^-1^), integrins α_2_β_1 _and α_IIb_β_3 _play a more significant role in platelet adhesion and recruitment. These platelet integrins are essential for thrombus formation at intermediate to high shear [26]. Furthermore, these integrins must become activated, that is convert into a high affinity conformation, in order for them to be capable of supporting adhesion at these high levels of shear [27]. This requires intracellular signal transduction events and platelet activation, a process that is inhibited by nitric oxide. Our results (Figures 1A and 1B) suggest that insulin and, to a lesser extent, isoproterenol can both cause a decrease in the rate of growth of thrombi and that this decrease is concentration dependent. Furthermore, we showed that the mechanism of this inhibition of thrombus growth involves a nitric oxide/guanylyate cyclase pathway (Figures 2B and 4A).

Our results with the inhibitors of platelet signaling, L-NAME and ODQ, are consistent with previous studies. L-NAME completely abolished the anti-thrombotic effects of both insulin and isoproterenol at a shear rate of 1000 s^-1 ^showing that these agonists act through a pathway that involves nitric oxide. ODQ was effective in inhibiting the action of insulin, but was less effective in inhibiting the action of isoproerenol. This result is consistent with earlier findings that suggested that isoproterenol inhibited platelet activation partially through a mechanism involving adenylate cyclase [28] rather than guanylyate cyclase.

The results we present with the inhibitors L-NAME and ODQ at the highest shear rate, 2000 s^-1 ^(Figures 2C, 4C, and 4D) failed to demonstrate a role for platelet-derived NO or signaling through guanylyate cyclase. However, in the absence of added agonist, shear stress, by itself was able to induce platelets to produce NO (Figures 3C and 5B). Although there has been no previous evidence for shear-dependent NO production in platelets, both *in vitro *and *in vivo *studies have demonstrated shear stress upregulates eNOS activity in endothelial cells [7,29]. The mechanism is not completely understood but involves cytoskeletal proteins and the activation of a series of kinases including PI3 kinase and Akt as well as Hsp90 [30,31]. A similar pathway for the activation of eNOS has been recently characterized in platelets in response to insulin [8].

## Conclusions

Our study focused on quantifying the effect of platelet-derived NO on platelet adhesion and aggregation on a surface in response to external factors (i.e. insulin, isoproterenol, and shear) and in the presence of whole blood. Platelet-derived NO did not affect platelet adhesion at low shear but had a significant effect at intermediate and high shear. Production of NO in platelets was dominated by receptor-mediated interactions at intermediate shear and mechano-transduction at high shear. This study and future work may lead to a better understanding of platelet-derived NO and its role in healthy and diabetic vascular wound healing.

## Methods

### Materials

Low molecular weight heparin, HEPES, NaCl, collagen III from calf skin, bovine serum albumin (BSA), L-nitro-amine-methyl-ester (L-NAME), insulin from porcine pancreas, and isoproterenol were obtained from Sigma. Recombinant hirudin (r-hirudin) was obtained from Pentapharm and 1H-[1,2,4]oxadiazolo [4, 3-a]quinoxalin-1-one (ODQ) was obtained from Cayman Chemical. Mepacrine (quinacrine) was obtained from ICN Biomedicals. Fluorescein isothiocyanate (FITC) was generously donated by Paul Friese, University of Oklahoma Health Sciences Center, Oklahoma City, OK. The S12 anti-P-selectin monoclonal antibody was generously donated by Roger P. McEver, Oklahoma Medical Research Foundation, Oklahoma City, OK. Glass cover slips (24 × 50 mm) were obtained from Fisher Scientific.

### Preparation of Glass Coverslips

Each cover slip was washed with 20 mL nanopure H_2_O and 10 mL 95% ethanol. After washing was complete, the cover slips were then placed in a 95% ethanol bath for at least 12 hours. Before use, each cover slip was rinsed with an additional 20 mL 95% ethanol and allowed to air-dry for 15 minutes.

### Protein coating of Cover slips

Collagen III solution was prepared at 0.8 mg/mL in 17 mM acetic acid (pH 2.6) at least 24 hours prior to use. BSA solution was prepared at 0.1% in 10 mM HEPES/115 mM NaCl buffer (pH 7.4). Half of each cover slip was coated with collagen and allowed to incubate for 4 hours in a humidified environment (80–90%) at room temperature (~24°C). After incubation, each slide was rinsed with 10–15 mL of HEPES/NaCl buffer solution to remove excess collagen and coated with 0.1% BSA solution for at least 1 hour.

### Platelet Preparation

Venous blood collected from healthy donors (30–60 mL, depending on shear rate and length of experiment) was anticoagulated with low molecular weight heparin to a final concentration of 20 U/mL or r-hirudin to a final concentration of 40 anti-thrombin units/mL (ATU/mL). The fluorescent dye mepacrine was then added to a final concentration of 10 μM and allowed to incubate for 10–15 minutes. All donors gave informed consent to participate in our study according to methods approved by the University of Oklahoma Institutional Review Board.

### Flow Experiments

The arterial flow environment was modeled with an *in vitro *parallel plate flow chamber similar to those previously characterized [32,33]. The dimensions of the flow channel were the 0.013 × 1.3 cm for experiments done at a shear rate of 100 s^-1^, 0.013 × .8 cm for shear rate of 500 s^-1^, and 0.013 × .25 cm for shear rates of 1,000 and 2,000 s^-1^.

In some studies, 500 pM insulin or 100 μM isoproterenol was added to the anti-coagulated blood 10 minutes before the start of an experiment. In other studies, L-NAME, an inhibitor of platelet NO synthesis was added at a concentration of 200 μM for 20 minutes before the start of an experiment. In additional studies, ODQ, an inhibitor of guanylate cyclase was added at a concentration of 100 μM for 20 minutes before the start of an experiment [18,19,34]. In still other studies, combinations of L-NAME or ODQ and insulin or isoproterenol were added as described above, except that L-NAME or ODQ was added 20 minutes prior to insulin or isoproterenol addition. In each experiment, blood was perfused for 5 – 10 minutes.

In all studies, blood was used in experiments within two hours of collection. The order of experiments was varied randomly to insure that time-dependent platelet phenotype was not skewing the results. Additionally, flow cytometric studies of platelets showed no significant change in platelet activation, as measured by P-selectin expression, over the course of 4 hours (data not shown).

### Microscopy and Imaging Systems

A syringe pump provided the flow through the flow chamber. The flow chamber was placed on the stage of a Nikon Diaphot 300 inverted microscope. The illumination was provided by a 75W Xenon light source passing through a 480 nm excitation filter and a 40 × fluorite objective lens. The fluorescent emission from adherent platelets was passed through a 514 nm filter and converted to analog signal by an image intensifier and CCD camera. The image was recorded on VHS tape for subsequent analysis. The extent of mural thrombus formation on the surface was quantified in terms of the percent coverage of a representative area of the collagen-coated surface at the interface between the collagen and albumin. The analyzed area was approximately 100 μm wide by 150 μm in the direction of flow. Digital image analysis was performed on an SGI Indy workstation running the ISEE^® ^image analysis software by Inovision. A background image was acquired after blood began flowing over the surface but before adhesion of any platelets. This image was subtracted from subsequent images. Platelets were identified based on size and intensity using adjustable parameters. Images were acquired and analyzed every 10–30 seconds over the course of each experiment, which lasted from 5–10 minutes.

### Statistical Analysis

Statistical significance of the results was assessed with the aid of the software package SPSS (version 11.5 for Windows). The statistical tests used were the Post Hoc test for multiple comparisons and the student's t-test. Results were deemed significant if p < 0.05.

## Abbreviations

BSA, bovine serum albumin; cAMP, cyclic adenosine monophosphate; eNOS, endothelial nitric oxide synthase, FITC, fluorescein isothiocyanate; HEPES, N-2-hydroxyethylpiperazine-N-2-ethanesulfonic acid; L-NAME, L-nitro-amine-methyl-ester; NO, nitric oxide; ODQ, [1,2,4]oxadiazolo [4, 3-a]quinoxalin-1-one; PI3K, phosphatidylinositol-3-kinase; PRP, platelet rich plasma; vWF, von Willebrand factor

## Competing Interests

The authors declare that they have no competing interests.

## Authors' contributions

RW performed all of the experiments and MN conceived of the project and coordinated the data analysis. All authors read and approved the final manuscript.
